# Too much time? Time use and fertility-specific quality of life among men and women seeking specialty care for infertility

**DOI:** 10.1186/s40359-019-0312-1

**Published:** 2019-07-09

**Authors:** Rachel Cusatis, Nicole Fergestrom, Alexandra Cooper, Kate D. Schoyer, Abbey Kruper, Jay Sandlow, Estil Strawn, Kathryn E. Flynn

**Affiliations:** 1 Medical College of Wisconsin, 8701 W. Watertown Plank Rd, Milwaukee, WI 53226 USA; 2 Duke University, Box 90989, Durham, NC 27708 USA; 3 Advocate Aurora Health, 3289 N. Mayfair Rd, Wauwatosa, WI 53222 USA

**Keywords:** Infertility, Fertility-related quality of life, Anxiety, Time use, Decision making

## Abstract

**Background:**

There are known gender differences in the impacts infertility has on quality of life and well-being. Less is known about how infertile couples spend time on fertility-related tasks and associations with quality of life. The purpose of this study is to evaluate whether time spent on tasks related to family-building decision-making (including research, reflection, discussions with partner, discussions with others, and logistics) were associated with fertility-specific quality of life or anxiety among new patients.

**Methods:**

Couples or individuals (*N* = 156) with upcoming initial consultations with a reproductive specialist completed the Fertility Quality of Life (FertiQoL) tool, which produces a Core (total) score and four subscales: Emotional, Relational, Social, and Mind-Body. We developed questions to measure time spent in the previous 24 h on tasks related to family-building. We tested for differences by gender in time use (McNemar’s Test) and used ordinary least squares regression to analyze the relationship between time use and FertiQoL scores.

**Results:**

In the week before a new consultation, a higher percentage of women reported time spent in the past 24 h in research, reflecting, discussion with others, and logistics compared to male partners (all *p* < 0.05). In adjusted models, more time spent reflecting was associated with worse FertiQoL scores for both men and women, as well as with higher anxiety for men. Time spent in discussion with others was associated with higher anxiety for women but better Social FertiQoL scores for men.

**Conclusions:**

Couples seeking infertility consultation with a specialist reported spending time on tasks related to family-building before the initial visit. There were gender differences in the amount of time spent on these tasks, and time was associated with fertility-specific quality of life and anxiety.

## Background

The impact of infertility, which affects millions of people in the United States (US), extends beyond sexual or reproductive areas of life, with noted burdens on psychosocial well-being and quality of life [1–5]. Both men and women experiencing infertility have demonstrated higher rates of stress and decreased quality of life compared to their fertile counterparts [6, 7], in addition to potentially distressing experiences related to personal, marital, and social relationships [8, 9].

There are known gender differences in fertility-related quality of life among couples experiencing infertility [2, 10–14]. In general, women have higher perceived stress and lower quality of life during infertility compared to men experiencing infertility. Since infertility is the absence of a desired social role, psychological distress is also associated with infertility, with women again bearing a larger burden compared to men [15–17]. Furthermore, older age [10], lower educational attainment [2, 10, 11, 18, 19], and lower reported relationship satisfaction with a partner [10, 20] have been associated with reduced fertility-related quality of life. Yet, much of what is known about gender differences in fertility-related quality of life relies on retrospective data collected after specialty care for infertility. No US study to date has assessed fertility-specific quality of life prior to establishing treatment with a reproductive specialist [12, 19, 21]. Therefore, little is understood about fertility-specific quality of life among Americans preparing for their first consultation with a reproductive specialist, or potential differences between men and women. Infertility treatments are often lengthy, expensive, and emotional; understanding patients and their partner’s well-being before starting this process can provide valuable knowledge to clinicians who support couples and individuals in decision-making regarding family building.

Infertility has been shown to impact women’s mental health, with roughly 40% of women experiencing infertility reporting symptoms of anxiety or depression [22–25]. Research has shown that increased anxiety and depression during infertility has impacts on discontinuation of infertility care and pursuing specific types of treatment like in vitro fertilization (IVF) [22], while failed treatment has also shown to have implications on depression among women [23, 24]. Resolving infertility through assisted reproductive technology, alternative family-planning options (fostering, adoption), or choosing to live child-free, is likely to affect people in many ways, including how and when they spend their time on family-building tasks. Despite a growing interest in understanding the impact of infertility on mental and social health, little is understood about how time use relates to quality of life or mental health.

Though existing published literature does not speak directly to gender roles during infertility, gender differences in the distribution of time in parenting have shown to negatively impact maternal quality of life. Time use literature suggests mothers spend more time doing mental labor, including planning, scheduling, coordinating, and managing the events and activities for their families [26–29]. As a result, scholars argue that mothers are substantially more likely than fathers to feel overburdened with work and family responsibilities [29, 30]. The unequal time use in mental labor for women has been associated with negative emotional impact [28]. Moreover, it is well documented that women, overall, tend to ruminate and reflect more than men, which is associated with higher levels of depression [31]. Exploring whether there are gender disparities in time use and understanding the relationship between time use and fertility-specific quality of life, as well as general anxiety, will provide valuable additions to current understandings of quality of life and time use among couples experiencing infertility. Clinically, this information can help inform best practices for starting patients and partners on their path to parenthood by keeping in mind their day-to-day lives and any associations between time use and well-being.

The current study aims to investigate potential differences between men and women in the amount of time spent on various activities in connection with family-building, including researching options, personal reflection, discussing family-building with a partner, discussing family-building with others, and dealing with logistics. We then investigate the associations between time use and fertility-specific quality of life and general anxiety among men and women experiencing infertility, controlling for individual characteristics (e.g., age, educational attainment, race) and relationship satisfaction.

## Methods

### Study design and participants

At a Reproductive Medicine Center affiliated with a large academic medical center in Milwaukee, Wisconsin, a convenience sample of new patients was recruited between May 2013 and June 2014 through physician letters to 613 new patients who had upcoming initial consultations with a reproductive specialist. Of the patients who received the letter, 155 patients responded and were screened for eligibility including: (1) an initial appointment date scheduled at least 1 week in the future; (2) no previous children born through assisted reproductive technology,^1^ and (3) ability to communicate in English and provide informed consent. From these, 111 patients met inclusion criteria; 92 patients and 68 of their partners enrolled in the study. For the current analysis, we removed same sex couples (*n* = 4 individuals) in order to analyze gender differences by role within couples leaving 90 female patients and 66 male partners (*n* = 156). Each participant completed a self-administered questionnaire using Research Electronic Data Capture (REDCap) prior to the consultation with the reproductive specialist (median 3 days prior to consultation; interquartile range = one to 6 days).

### Measures

The primary variables of interest for this study were self-reported fertility-specific quality of life, general anxiety, and time spent on activities pertaining to the family-building decision-making process.

The FertiQoL tool assesses fertility-specific quality of life in people experiencing infertility [29, 30] with evidence for reliability and validity in national and international populations, including Cronbach’s alphas ranging from 0.72 to 0.92 [33, 34], as well as omegas with 95% confidence interval (CI) in our sample for Core (0.90; CI 0.87–0.92), Emotional (0.90; CI 0.87–0.92), Relational (0.90; CI 0.88–0.92), Social (0.90; CI 0.87–0.92), Mind/Body (0.90; CI 0.87–0.92). Four 6-item subscales comprise the Core (total) scale: Emotional (fertility-related negative emotions such as jealousy, resentment, sadness, and depression), Relational (fertility-related problems within one’s marriage or partnership including communication, commitment, and sexuality), Social (the extent to which social interactions such as social inclusion, expectations, stigma, and support have been affected by fertility problems), and Mind-Body (negative physical, cognitive, or behavioral symptoms related to infertility). The FertiQoL tool has a range of 0–100. A higher score indicates better fertility quality of life.

We used the Patient-Reported Outcomes Measurement Information System (PROMIS) Anxiety short form 4a to measure self-reported anxiety [32]. Scores are reported on the T metric, and a score of 50 (SD of 10) corresponds to the US general population (Cronbach’s alpha = 0.93 in a national sample and omega with 95% CI within our sample = 0.85; CI 0.80–0.90) [35]. Higher values indicate higher levels of anxiety. Normative anxiety scores for women are slightly higher than men (50.9 for women compared to 48.6 for men). Further, those between the ages of 18–34 have higher average anxiety (52.4) compared to older individuals (age 34–44: 50.9) [36].

Using the FertiQoL and PROMIS Anxiety instruments allows us to speak to the previous literature on well-being and anxiety among infertility patients and contribute to the literature by adding partner’s well-being and anxiety.

We created five items to assess time spent on fertility-related activities. To evaluate content and face validity, these items were tested in 17 cognitive interviews (10 women, 7 men) with people recruited from the same fertility clinic who had undergone assisted reproductive technology. Participants were asked to report the amount of time in the past 24 h they spent in research such as “looking for information” and “researching my options,” personal reflection, discussing with their partner, discussing with others, and on logistics such as calling about insurance coverage or ordering prescriptions using a 5-category response system (“no hours”, “less than one hour,” “1–2 h,” “3–4 h,” and “5 or more hours”); for exact wording see the Supplemental Appendix A. These items were designed to measure time spent on various tasks during treatment for infertility, thus at this initial data collection point before commencing treatment, data were sparse in many categories. Accordingly, we collapsed response options for analysis into dichotomous indicators of whether or not someone dedicated any time to each family-building task (no time vs. any time). One exception was reflection among women, which had more variability and which we therefore collapsed into three categories: less than 1 hour (38%), 1–2 h (28%), and 3+ hours (34%). Sociodemographic characteristics previously shown in the literature to impact fertility-specific quality of life were included as covariates. Age was included as a continuous variable. Education was operationalized as a dichotomous variable indicating whether a patient attained a college degree (less than college vs. college or more). Race/ethnicity was dichotomized (non-Hispanic white vs. other race/ethnicity). Finally, we included the 4-item Couples Satisfaction Index (0–21) to measure relationship satisfaction where higher scores indicate greater satisfaction in one’s relationship, with previously recommended distress cut off of 13.5 and high internal consistency (Alpha coefficient = .94) [37].

### Analyses

Survey responses were exported from REDCap to STATA (version 14.1) and R (version 3.4.3). Statistical analyses used were percentage, frequency, McNemar’s test, and ordinary least squares (OLS) regression. Percentage, frequency, and McNemar’s tests were calculated in R, and OLS was analyzed in STATA. In addition to providing information on the sample, descriptive statistics for the full sample and by gender provided initial evidence of the ways in which men and women differ on their time spent in family-building tasks, FertiQoL, and anxiety. We describe these differences among all men and women first, and second, we use McNemar’s Test within the smaller subsample of couples to statistically test for differences in time spent by gender, FertiQoL, and anxiety. Finally, we used separate regressions for men and women to evaluate whether time spent on family-building tasks impacted FertiQoL or anxiety scores, controlling for known correlates.

### Ethics approval and consent to participate

The research was approved for scientific and ethical integrity by the Institutional Review Board of the Medical College of Wisconsin. All participants provided written or electronic informed consent.

## Results

### Socio-demographic characteristics of study participants

In general, the sample was highly educated (73% have college degree or more), with 85% identifying as non-Hispanic white, and a mean age of 34 years (Table 1). Both men and women reported about an average time of 2 years (24 months) trying to conceive. The women on average reported higher education and were younger than the men. Prior to their initial consultation, women reported slightly lower FertiQoL scores than men across all subscales, indicating lower fertility-specific quality of life. The difference in scores was small for the Relational and Social subscales but notable for the Emotional subscale. Likewise, women reported slightly higher general anxiety compared to men, consistent with US norms [36].

**Table 1:** Sample Characteristics (*n* = 156)

Percentages		
	Women (*N* = 89)	Men (*N* = 67)
FertiQoL		
*Core, mean (SD)*	68.9 (15.4)	79.7 (12.0)
*Emotional, mean (SD)*	55.4 (21.7)	78.3 (17.9)
*Social, mean (SD)*	70.8 (17.9)	75.7 (16.5)
*Mind Body, mean (SD)*	75.1 (19.6)	88.8 (12.6)
*Relational, mean (SD)*	74.4 (16.6)	76.1 (14.5)
PROMIS Anxiety*, mean (SD)*	50.6 (8.0)	48.7 (7.9)
Time Spent on:		
*Research*		
No time	46.1	68.7
Any time	53.9	31.3
*Personal Reflection*		
No time	7.9	26.9
Any time	92.1	73.1
*Discussion with Partner*		
No time	16.9	16.4
Any time	83.2	83.6
*Discussion with Others*		
No time	31.5	64.2
Any time	68.5	35.8
*Logistics*		
No time	61.8	83.6
Any time	38.2	16.4
Demographics		
*Time Trying* ^*1*^	24.7 (28.6)	22.2 (16.9)
*Couples Satisfaction Index* ^*2*^ *, mean (SD)*	17.7 (2.5)	17.4 (2.9)
*Education*		
Less than College	21.6	34.4
College Degree +	78.4	65.6
*Age, mean (SD)*	32.8 (5.2)	35.5 (6.7)
*Race/Ethnicity*		
Non-Hispanic White	84.3	85.1
Hispanic and non-White	15.7	14.9

^1^Time Trying was self-reported time (in months) trying to conceive a child; ^2^Couples Satisfaction Index ranges from 0 to 21

Table 1 shows time spent in discussions about family-building with one’s partner were remarkably similar between women and men, with 83.2% of women and 83.6% of men reporting time spent on this activity during the previous 24 h. Differences between men and women in time use were seen in the other four categories. Over 50% of women reported engaging in some research in the 24 h prior while only 31% of men did. For time spent on personal reflection, 92% of women and 73% of men reported at least some time reflecting in the previous 24-h. More women (68%) than men (36%) indicated spending time discussing family-building with others. Finally, few participants report any time on logistics, though more women (38%) report at least some time on logistics compared to men (16%).

### Within couple differences in time spent on family-building tasks

The McNemar’s tests demonstrate that men and women differ on the amount of time spent on family-building decision-making tasks (Table 2). The patterns seen within couples are the same as described in the full sample; time spent on family-building tasks is significantly different for men and women within couples for all tasks except discussion with partner. A larger percentage of women compared to their male partners reported time spent: researching – 19.7 percentage point difference favoring women, reflecting − 18.2 percentage points, discussing with other people – 31.8 percentage points, and logistics – 15.1 percentage point difference.

**Table 2:** Within couple differences in time spent on family-building tasks (*n* = 132 individuals, 66 couples)

Time Use	% Women Reporting Any Time	% Men Reporting Any Time	McNemar’s Chi-Square	*p*-value
*Research*				
No time v. any time	50	30.3	4.65	.031
*Reflecting*				
No time v. any time	90.9	72.7	7.56	.006
*Discussion w/Partner*				
No time v. any time	81.8	83.3	0	1
*Discussion in General*				
No time v. any time	68.2	36.4	11.4	.0007
*Logistics*				
No time v. any time	31.8	16.7	4.5	.034

### Time use and quality of life

We used regression analysis to understand whether time spent in family-building decision-making tasks was associated with fertility-specific quality of life or anxiety separately among women (Table 3) and men (Table 4).

**Table 3:** OLS Linear Regression Models Predicting Fertility-Specific Quality of Life (FertiQoL) and Anxiety for Women (*n* = 88)

	(A) FertiQoL Core		(B) FertiQoL Emotional		(C) FertiQoL Social		(D) FertiQoL Mind/Body		(E) FertiQoL Relational		(F) PROMIS Anxiety	
	Beta	Std. Err.	Beta	Std. Err.	Beta	Std. Err.	Beta	Std. Err.	Beta	Std. Err.	Beta	Std. Err.
Time Use (no time spent)												
*Research*	−.575	3.53	1.92	5.12	−1.62	3.52	−5.25	4.89	3.80	3.36	1.50	2.21
*Reflecting* (less than 1 hour)												
*1–2 hours*	− 4.9	4.12	−6.49	5.98	− 11.4*	5.28	1.84	5.71	−3.54	3.91	.909	2.58
*3+ hours*	−9.5*	4.20	−14.0*	6.10	− 12.3*	5.39	−5.82	5.83	−4.96	4.03	−.518	2.63
*Discussion with partner*	.332	4.01	.489	5.82	−.435	5.14	2.19	5.55	−2.64	4.03	−4.13	2.51
*Discussion with others*	−1.73	3.32	−6.58	4.82	−1.73	3.16	− 6.08	4.60	.159	3.15	4.18*	2.07
*Logistics*	−3.03	3.26	−8.45	5.83	−1.73	4.18	1.20	4.52	−2.67	3.11	−.750	2.04
Covariates												
*CSI^*	1.83**	.547	1.06	.794	.890	.702	1.01	.758	4.34***	.519	−.185	.342
*College degree or more* vs. *less*	3.50	3.35	4.31	4.87	5.77	4.31	4.33	4.65	−.089	3.19	2.55	2.10
*Age*	1.11***	.292	1.35**	.424	.864*	.702	1.44**	.405	.740*	.278	−.181	.183
*Non-White vs. Non-Hispanic White*	1.21	4.01	8.01	5.83	−3.82	5.15	1.31	5.57	−.829	3.82	2.82	2.51

^Couples Satisfaction Index (CSI)

*p < .05; ** < .01; *** < .001

**Table 4:** OLS Linear Regression Models Predicting Fertility-Specific Quality of Life (FertiQoL) and Anxiety for Men (*n* = 64)

	(A) FertiQoL Core		(B) FertiQoL Emotional		(C) FertiQoL Social		(D) FertiQoL Mind/Body		(E) FertiQoL Relational		(F) PROMIS Anxiety	
	Beta	Std. Err.	Beta	Std. Err.	Beta	Std. Err.	Beta	Std. Err.	Beta	Std. Err.	Beta	Std. Err.
Time Use (no time spent)												
*Research*	−2.24	2.94	−2.18	4.80	−5.61	4.07	−2.11	3.59	1.23	3.20	−2.14	2.39
*Reflecting*	−7.08*	3.41	−13.3*	5.57	−8.91	4.72	−6.53	4.17	.185	3.71	7.59**	2.77
*Discussion with partner*	−3.17	3.56	−6.38	5.82	−3.15	4.93	−2.44	4.35	−.589	3.88	1.36	2.90
*Discussion with others*	4.92	4.95	8.42	4.83	11.9**	4.09	1.05	3.61	−1.81	3.22	.489	2.41
*Logistics*	.300	3.65	−2.05	5.99	.494	5.07	−1.17	4.47	3.71	4.00	−1.43	2.98
Covariates												
*CSI^*	1.51*	.451	.237	.738	1.46*	.626	.443	.552	3.81***	.491	−.090	.368
*College degree or more* vs. *less*	−2.38	2.70	2.09	4.42	−9.17*	3.75	.246	3.30	−2.48	2.94	1.92	2.20
*Age*	.786***	.193	1.09**	.316	.998***	.268	.663**	.236	.374	.210	−.010	.157
*Non-White vs. Non-Hispanic White*	−4.45	4.15	3.19	6.79	−16.7**	5.75	−2.02	5.07	−2.35	4.52	−.576	3.38

^Couples Satisfaction Index (CSI)

*p < .05; ** < .01; *** < .001

### Women’s fertility-specific quality of life and anxiety

The first column in Table 3 (A) reports unstandardized coefficients for the relationships between each variable and the composite FertiQoL Core total score for women. Those who spent 3 h or more reflecting had significantly lower fertility-specific quality of life compared to women who did not engage in any reflection (9.5 points lower). No other time-use variables were significantly related to Core FertiQoL. Higher Couples Satisfaction Index scores and older age were positively related to women’s FertiQoL Core, meaning those women with better relationship satisfaction and those who were older had better overall fertility-specific quality of life.

Column (B) in Table 3 shows unstandardized coefficients among women for the relationships between each variable and the FertiQoL Emotional subscale (i.e., negative emotions). We see similar results to Core FertiQoL, women spending three or more hours reflecting had significantly lower reports of Emotional FertiQoL compared to women who did not spend any time reflecting about family-building – 14 points less. Finally, age was significantly related to Emotional FertiQoL scores with older women reporting significantly better scores.

Column (C) in Table 3 shows relationships among women between each variable and the FertiQoL Social subscale (i.e., social inclusion, expectations, stigma, support). Time spent in personal reflection about family-building demonstrated a significant relationship with fertility-specific social quality of life. Spending any time reflecting was associated with a reduction in women’s Social FertiQoL: those women spending one to 2 hrs reflecting reported scores 11.4 lower and those spending three or more hours reflecting had scores 12.3 points lower compared to women who did not spend any time in reflection. Again, older women reported significantly better Social FertiQoL scores compared to younger women.

Columns (D and E) report results for the Mind/Body and Relational subscales among women, respectively. For both subscales, none of the time use variables demonstrate significant associations with FertiQoL. However, age was associated with Mind/Body and Relational scores indicating that older women reported significantly better FertiQoL scores compared to younger women. For Relational FertiQoL, those with higher couples’ satisfaction reported significantly better Relational scores.

The final column in Table 3 (F) reports relationships between each variable and women’s self-reported general anxiety. For women, time spent engaging in discussions about family-building with people other than their partner was associated with higher anxiety (4.2 points) compared to women who did not report spending time in this way. No other variables in the model were associated with anxiety scores.

### Men’s fertility-specific quality of life and anxiety

The first column (A) in Table 4 illustrates relationships between time spent on family-building tasks, sociodemographic characteristics and overall fertility-specific quality of life (FertiQoL Core). Men who spent any time in personal reflection about family-building reported significantly lower fertility-specific quality of life (7 points). Men with higher reported relationship satisfaction reported significantly better Core FertiQoL scores. Older men also had significantly better fertility-specific quality of life.

The next model (B) assessed the relationships between time spent on different family-building decision-making tasks and the Emotional FertiQoL subscale. Again, men who reported any time spent in personal reflection had significantly lower (13 points) scores on the Emotional subscale of the FertiQoL. Time spent on other tasks did not significantly relate to men’s Emotional FertiQoL. Consistent with women, older age was positively related to better emotional fertility-specific quality of life.

The third column in Table 4 (C) documents unstandardized coefficients for the relationships between each variable and the FertiQoL Social subscale for men. Men who spent time discussing family-building with someone other than their partner had significantly lower Social FertiQoL scores, by about 12 points. Time spent in research, reflections, discussions with partner, or logistics did not significantly impact Social FertiQoL. Older men and those who reported higher relationship satisfaction had significantly higher Social FertiQoL scores. Conversely, for men, having a college degree or more was significantly associated with lower Social FertiQol scores by about 9 points compared to having with less education, and identifying as non-white was associated with lower scores (16 points) on Social FertiQoL compared to identifying as white, non-Hispanic.

Similar to women, men’s Mind/Body (D) and Relational (E) scores did not demonstrate significant associations with any time use variables. For Mind/Body, older men reported significantly better Mind/Body FertiQoL. For Relational, men with higher couples’ satisfaction reported significantly better Relational FertiQoL.

Results for men’s anxiety are recorded in the final column (F). Men who spend any time reflecting on family-building reported significantly higher anxiety compared to men who did not spend any time reflecting (7.6 points). No other time use tasks or demographic characteristics were associated with anxiety scores for men.

## Discussion

Treatment, management, and decision-making in infertility can be extensive for couples, and time is a finite commodity. Research has demonstrated that the ways men and women spend time is significantly different, and this has implications for their health. In this study, we describe the time spent on various activities related to family-building decision-making for men and women who have made an appointment for a new consult with a reproductive specialist but have not yet had the appointment or started fertility treatments. In the previous 24 h, about half of women and 1/3rd of men had spent time looking for information or researching options to grow their family. Nearly all women and 3/4^ths^ of men had spent time in personal reflection. Over 80% of men and women had spent time discussing family-building with their partner, while 2/3^rd^^s^ of women but only 1/3^rd^ of men had discussed family-building with people other than their partner. About 1/3^rd^ of women but only 16% of men had spent time dealing with logistics related to family-building.

We described the fertility-specific quality of life and general anxiety reported by men and women in this US-based sample, which is unique for its inclusion of male partners and the timing of the assessment (in the days leading up to an initial specialty consultation). We observed gender differences in: (1) the amount of time spent on fertility-related decision-making tasks within couples experiencing infertility, and (2) how time spent on different tasks is associated with fertility-specific quality of life and general anxiety. Previous research consistently shows that women ruminate and reflect more than men [30] and that gender imbalances in time spent on family tasks has been shown to lead to negative health outcomes [28, 31]. Our data was consistent with this; significantly more women compared to their male partners spent time in the previous 24 h in personal reflection about family-building. For both men and women, this time spent was associated with negative emotions specific to infertility, but for men it was also associated with higher general anxiety. The experience of infertility likely affects men and women differently, which may be due in part to differences in time use. When it comes to rumination and reflection, we observed differences between men and women in the relationships between these activities and fertility-specific quality of life. It is possible that rumination and reflection may serve as more of an interruption in men’s lives than women’s, which is associated with higher anxiety. Clinically, these differences may be important when supporting couples with the psychological aspects of infertility - ensuring each has realistic expectations of their partner with how time is spent and task completion. For example, women may desire a greater amount of verbal discussion and reflection regarding infertility diagnosis and treatment. However, if men perceive this as anxiety-inducing they may be less inclined to engage. This incongruence between needs and experiences may be a dynamic that affects couples’ communication, coping, and navigation around fertility-related issues. Previous research on dyadic coping recognizes that when men and women within a couple employ different coping strategies, distress and depression are impacted for both members of the couple [38, 39]. For example, when men use distancing to cope and their partners do not, both members of the couple experience increased distress [39]. Clinicians can support couples by identifying what each expects, desires, and needs regarding time spent in discussion and task completion. Further, paternal and maternal well-being and anxiety are known to be related, therefore aligning partner expectations—or addressing partner differences—may help reduce these anxieties [40]. This hypothesis could be further explored in other samples and settings.

These results also show a key factor to improving men’s social quality of life was discussing their infertility with others. Previous studies observed men more often choose not to share their infertility experience with anyone other than their partner, and the lack of openness with others was predictive of depression [41, 42]. Our findings align with this prior work, demonstrating that when men *do* open up to others, it is associated with improved quality of life. This provides evidence of the importance of screening for and discussing the importance of social support during infertility for not only women, but men as well. Clinically, men may benefit from education on the role of social support for men going through infertility. Educating them on the research, benefits, and types of support may facilitate or increase their willingness to obtain social support that can act as a buffer for distress and coping.

A consistent finding across the different models was that men and women who were older reported better well-being, regardless of time spent on family building tasks. This may be a reflection of the overall trends in mental health improving with age [43]. Alternatively, or additionally, patients who are seeking specialty care for infertility at a later age have lived a longer time child-free or may already have children, which may make it easier to cope with infertility. Additionally, older couples may not have as many peers conceiving and thus may experience fewer social reminders of their own infertility, e.g., attending friends’ baby showers.

This study provides unique insight into the intersection of time spent on family-building tasks, fertility-specific quality of life, anxiety, and gender differences. We provide one of the first investigations into an American sample of patients *prior* to their appointment with a reproductive specialist. Further, analysis of both men’s and women’s experiences with infertility as they relate to quality of life and anxiety is also an important contribution. Despite these strengths, we also recognize limitations in this study and sample. The cross-sectional design renders it impossible to draw conclusions about causality, and we are unable to determine whether time spent on these activities causes lower quality of life or vice versa. Our findings do, however, identify important associations between the two. This study made use of convenience sampling at one suburban academic medical center with relatively low response rate (25.3%), and the time use and gender differences of participants may differ from non-respondents both within the academic medical center and the general population more broadly, including individuals who experience infertility but do not seek consultation from a specialist, rendering findings potentially subject to selection bias. Additionally, the sample size is relatively small, which potentially reduces observed variability in time use and may impact our ability to detect significant associations. Finally, the time use questions asked about the previous 24-h. We chose this recall period to reduce concerns about recall bias, but the short time frame may miss time spent on family building tasks over a longer time period.

## Conclusions

For men and women, more time spent in reflection was associated with lower fertility-specific quality of life; moreover, for men it was also associated with higher anxiety. Time spent discussing family-building with people other than their partner was associated with higher anxiety for women, yet for men this activity was associated with better fertility-specific social quality of life. This data supports the importance of screening patients’ emotional and psychological status at initial consultation with an infertility provider, as patients’ baseline status is predictive of subsequent anxiety and depression during ongoing evaluation and treatment.

## Data Availability

The study is part of a larger on-going study and therefore we are not able to make our data public.

## Abbreviations

CI: Confidence Interval
FertiQoL: Fertility-specific Quality of Life tool
IVF: in-vitro fertilization
OLS: Ordinary Least Squares
PROMIS: Patient-Reported Outcomes Measurement Information System
REDCap: Research Electronic Data Capture
US: United States

## Footnotes

1 Importantly, this does not mean all participants were childless. Some of our participants previously had children, most often from relationships with other partners.
